# RNA Sequencing of Primary Cutaneous and Breast-Implant Associated Anaplastic Large Cell Lymphomas Reveals Infrequent Fusion Transcripts and Upregulation of PI3K/AKT Signaling via Neurotrophin Pathway Genes

**DOI:** 10.3390/cancers13246174

**Published:** 2021-12-07

**Authors:** Arianna Di Napoli, Davide Vacca, Giorgio Bertolazzi, Gianluca Lopez, Maria Piane, Aldo Germani, Evelina Rogges, Giuseppina Pepe, Fabio Santanelli Di Pompeo, Marzia Salgarello, Vaidehi Jobanputra, Susan Hsiao, Kazimierz O. Wrzeszczynski, Emilio Berti, Govind Bhagat

**Affiliations:** 1 Department of Clinical and Molecular Medicine, Sant’Andrea Hospital, Sapienza University, 00189 Rome, Italy; glopez@ospedalesantandrea.it (G.L.); maria.piane@uniroma1.it (M.P.); aldo.germani@uniroma1.it (A.G.); evelina.rogges@uniroma1.it (E.R.); giuseppina.pepe@uniroma1.it (G.P.); 2 Department of Surgical, Oncological and Oral Sciences, Palermo University, 90134 Palermo, Italy; davide.vacca@unipa.it; 3 Tumour Immunology Unit, Human Pathology Section, Department of Health Science, Palermo University, 90134 Palermo, Italy; giorgio.bertolazzi@unipa.it; 4 Plastic Surgery Unit, Sant’Andrea Hospital, Sapienza University, 00189 Rome, Italy; fabio.santanelli@uniroma1.it; 5 Department of Plastic Surgery, Catholic University of Sacred Heart, University Hospital Agostino Gemelli, 00168 Roma, Italy; marzia.salgarello@policlinicogemelli.it; 6 Department of Pathology and Cell Biology, Columbia University Medical Center, New York Presbyterian Hospital, New York, NY 10032, USA; vjobanputra@nygenome.org (V.J.); sjh2155@cumc.columbia.edu (S.H.); gb96@cumc.columbia.edu (G.B.); 7 New York Genome Center, New York, NY 10013, USA; kwrzeszczynski@nygenome.org; 8 Department of Dermatology, Fondazione IRCCS Cà Granda Ospedale Maggiore Policlinico, 20122 Milan, Italy; emilio.berti@unimi.it

**Keywords:** ALCL, fusion transcripts, transcriptome, PI3K/Akt pathway, NTRK signaling

## Abstract

**Simple Summary:**

Cutaneous and breast implant-associated anaplastic large-cell lymphomas are usually localized neoplasms with an indolent clinical course compared to systemic ALCL. However comparative analyses of the molecular features of these two entities have not yet been reported. We performed targeted RNA sequencing, which revealed that fusion transcripts, although infrequent, might represent additional pathogenetic events in both diseases. We also found that these entities display upregulation of the PI3K/Akt pathway and show enrichment in genes of the neurotrophin signaling pathway. These findings advance our knowledge regarding the pathobiology of cALCL and BI-ALCL and point to additional therapeutic targets.

**Abstract:**

Cutaneous and breast implant-associated anaplastic large-cell lymphomas (cALCLs and BI-ALCLs) are two localized forms of peripheral T-cell lymphomas (PTCLs) that are recognized as distinct entities within the family of ALCL. JAK-STAT signaling is a common feature of all ALCL subtypes, whereas DUSP22/IRF4, TP63 and TYK gene rearrangements have been reported in a proportion of ALK-negative sALCLs and cALCLs. Both cALCLs and BI-ALCLs differ in their gene expression profiles compared to PTCLs; however, a direct comparison of the genomic alterations and transcriptomes of these two entities is lacking. By performing RNA sequencing of 1385 genes (TruSight RNA Pan-Cancer, Illumina) in 12 cALCLs, 10 BI-ALCLs and two anaplastic lymphoma kinase (ALK)-positive sALCLs, we identified the previously reported TYK2-NPM1 fusion in 1 cALCL (1/12, 8%), and four new intrachromosomal gene fusions in 2 BI-ALCLs (2/10, 20%) involving genes on chromosome 1 (EPS15-GNG12 and ARNT-GOLPH3L) and on chromosome 17 (MYO18A-GIT1 and NF1-GOSR1). One of the two BI-ALCL samples showed a complex karyotype, raising the possibility that genomic instability may be responsible for intra-chromosomal fusions in BI-ALCL. Moreover, transcriptional analysis revealed similar upregulation of the PI3K/Akt pathway, associated with enrichment in the expression of neurotrophin signaling genes, which was more conspicuous in BI-ALCL, as well as differences, i.e., over-expression of genes involved in the RNA polymerase II transcription program in BI-ALCL and of the RNA splicing/processing program in cALCL.

## 1. Introduction

Anaplastic large-cell lymphomas (ALCLs) are a heterogeneous group of peripheral T-cell lymphomas (PTCLs) differing in their sites of occurrence, prognosis and molecular signatures. Cutaneous (cALCLs) and breast implant-associated anaplastic large cell lymphomas (BI-ALCLs) are usually indolent, presenting as localized diseases [1], whereas the systemic form manifests as an aggressive disease, with enlarged lymph nodes and less frequently extranodal involvement. The presence or absence of anaplastic kinase lymphoma (ALK) gene translocations is further used to categorize sALCLs as ALK-positive sALCLs and ALK-negative sALCLs.

BI-ALCLs and cALCLs share the activation of the JAK/STAT3 pathway with other PTCLs [2,3,4,5,6,7], but they have distinct gene expression profiles. In particular, in BI-ALCLs upregulated programs include transcription, migration and myeloid cell differentiation, whereas T-cell activation and the immune response are downregulated [7]. Compared to the ALK-DUSP22-TP63-triple negative ALCL, BI-ALCLs also show upregulation of hypoxia signaling genes [8]. The cALCL transcriptome differs from that of PTCL-NOS, exhibiting higher expression of the skin-homing chemokine receptor genes CCR10, CCR8 and CCR7; the MET gene that encodes the hepatocyte growth factor receptor and genes involved in apoptosis (TNFRSF8/CD30, JMY, RFFL, TMEM23/SGMS1, TRAF1, HIP1, PMAIP1 and CDKN2C/p18) [9].

Molecular studies have identified rearrangements of the DUSP22/IRF4 locus on chromosome 6p25.3 in approximately 30% of ALK-sALCLs and cALCLs [10,11,12,13,14], and of the TP63 gene on chromosome 3q26.92 in about 9% of ALK-sALCLs and in 5% of cALCLs, with the TP63-rearranged cases showing worse prognosis [13,14,15,16,17]. In contrast, FISH analysis has failed to detect both DUSP22 and TP63 translocations in BI-ALCLs [18,19]. Other recurrent alterations in cALCLs include JUNB amplification (70% of cases) [20,21], TYK2 rearrangement (4/32, 12.5% of cases) [5], and gains on chromosome 7q and losses on 6q and 13q (altogether accounting for 45% of cases) [9]. BI-ALCLs, on the other hand are characterized by frequent losses on chromosome 20q13.13 (66% of cases) [22], PD-L1 locus amplification at 9p24.1 (33% of cases) [23] and by recurrent mutations in JAK/STAT signaling genes, epigenetic modifiers and TP53 [24,25,26,27,28].

All the studies performed thus far have contributed important pieces of information regarding the molecular aspects of cALCL and BI-ALCL; however, the lack of a direct comparison of the two diseases limits our comprehension of pathobiological similarities and differences. To investigate the relationship between BI-ALCL and cALCL and detect gene fusions, we applied an RNA sequencing (RNA-seq) approach, investigating 1385 genes recurrently translocated, mutated or deregulated in cancer, in 12 cALCLs, 10 BI-ALCLs and 2 ALK+ sALCLs. Our findings provide additional insights into the transcriptional profiles of cALCLs and BI-ALCLs, confirms the rarity of gene rearrangements in these diseases, and uncovers novel intrachromosomal gene fusions in BI-ALCLs.

## 2. Materials and Methods

### 2.1. Sample Collection

Formalin-fixed paraffin-embedded (FFPE) tissue samples from 12 cALCLs, 10 BI-ALCLs and 2 anaplastic lymphoma kinase (ALK)-positive systemic ALCLs (ALK+ sALCLs) were collected. The BI-ALCLs and ALK+ sALCLs were diagnosed at Sant’Andrea Hospital of Rome, whereas cALCLs were diagnosed at Policlinico di Milano Ospedale Maggiore|Fondazione IRCCS Ca’ Granda. The research was performed in accordance with the Declaration of Helsinki and this study was approved by the Ethics Committee of Sant’Andrea Hospital/University “Sapienza” of Rome (EC n. 82 SA_2017 and EC n. 198 SA_2021).

### 2.2. Laser Microdissection and RNA Extraction

To enrich for BI-ALCL tumor cells and minimize contamination by RNA derived from non-neoplastic stromal and inflammatory cells, we performed laser microdissection of FFPE tissue sections using the Laser Microdissector SL CUT (Nikon Instruments, New York, NY, USA) as previously described [7]. Total RNA was then extracted and purified using a High Pure miRNA Isolation Kit (Roche, Basel, Switzerland) from a total of ~3000 microdissected tumor cells per BI-ALCL and from whole-tissue sections of cALCL and ALK+ sALCL samples with high tumor content, according to the manufacturer’s instructions. RNA quality was evaluated by measuring RIN (RNA Integrity Number) and DV200% (% Distribution value of fragments ≥200 nucleotides) parameters using the Bioanalyzer 2100 System (Agilent Technologies, Santa Clara, CA, USA).

### 2.3. RNA Sequencing

To detect fusion transcripts in ALCLs, the TruSight RNA Pan-Cancer panel (Illumina, San Diego, CA, USA) targeting 1385 cancer genes, including 507 known genes involved in fusions and 878 genes either mutated or deregulated in cancers, was used according to the provided protocol. The panel design covers all exons and 160 bp at the 5′ and 3′ UTR of every gene included in the panel. Briefly, double-stranded cDNA fragments generated from 50 ng of total RNA were ligated to sequencing adapters. The coding regions of expressed cancer-associated genes were captured using sequence-specific probes to create the final sequencing library. Paired-end RNA-sequencing was performed on a NextSeq500 sequencer using the NextSeq500 High Output Kit v2 chemistry (Illumina). Raw sequencing data converted to fastq file formats were analyzed.

Sequencing data are available at the Sequence Read Archive (https://www.ncbi.nlm.nih.gov/sra (accessed on 1 December 2021)).

### 2.4. RNA-Sequencing Data Analysis and Fusion Detection

The presence of fusion gene transcripts was analyzed by two computational tools: FusionCatcher and FusionMap on the RNA-seq data as previously described [29,30] FusionCatcher aligns the sequencing reads, as single reads, of transcriptomes using Ensembl genome annotation and the Bowtie aligner, whereas Fusionmap alignment is an implementation of a modified GSNAP method [31]. The TruSight RNA Pan-Cancer panel through its oligo capture approach enables pulling down one target gene among the 1385 genes in the panel, as well as the fusion partner not necessarily included in the panel. Only fusion transcripts identified by both methods were further analyzed.

### 2.5. Confirmation of Novel Fusions by Real-Time Polymerase Chain Reaction Analysis

In order to validate the presence of fusion transcripts, real-time quantitative polymerase chain reaction (RT-PCR) was performed with a Rotor-Gene Q system (Qiagen, Hilden, Germany.) using the Quantinova Probe RT-PCR kit (Qiagen, Hilden, Germany) according to the manufacturer’s instructions. Parallel amplification reactions were carried out using TaqMan gene expression assays. All reactions included two hold thermal steps to 45 °C for 10 min and 95 °C for 5 min, followed by 40 cycles of denaturation and annealing/extension at 95 °C for 5 s and at 60 °C for 30 s, respectively, except when analyzing the NPM-TYK2 fusion transcript, where the annealing temperature was set at 55 °C for 30 s. Assays were performed in duplicate instead of triplicate due to the low amount of RNA obtained from microdissected samples. Primers and probes for RT-qPCR assays of NPM1-TYK2, MYO18A-GIT1 and GOSR1-NF1 fusion transcripts were designed using the Primer3Plus tool [32] (Table 1), whereas the Custom TaqMan^®^ Assay Design Tool by ThermoFisher Scientific, was used to design the assays for GOLPH3L-ARNT (Assay ID: ARPRMEK) and EPS15-GNG12 (Assay ID: ARPRMEK) fusion transcripts. All forward primers and probes were designed to recognize the 5′-3′ strand upstream and downstream of the breakpoint, whereas reverse primers were designed to recognize the 3′-5′ strand downstream of the breakpoint. ACTb was used as an endogenous control (ThermoFisher Scientific, product ID: Hs01060665_g1). All gene target probes were conjugated with FAM, whereas the probe for the endogenous control was conjugated with a VIC fluorophore.

### 2.6. Differential Gene Expression Analysis

RNA seq data in fastq format were aligned to the human genome *GRCh38.99* using STAR (Spliced Transcripts Alignment to a Reference) [33]. Starting from the bam files obtained from the STAR output, we performed read counting using the function summarizeOverlaps from the GenomicAlignmentsR package [34]. After running a correlation analysis over read lanes, those that referred to the same sample were unified using average counting. Read counts were converted to log2-counts-per-million using the cpm function of the R limma package [34].

All statistical analyses were performed using R statistical software v4.0.2 (http://www.R-project.org (accessed on 7 September 2021)). The Euclidean distance metric across the 24 normalized samples (12 BI-ALCLs, 10 cALCLs, and 2 ALK+ sALCLs) was considered for hierarchical clustering analysis, and the *complete* aggregation method was used to build the heatmap dendrogram within the R package heatmap. Principal component analysis was performed using the FactoMineR R package.

Differential expression analysis between BI-ALCL and cALCL samples was carried out by applying the moderated t-test using the limma package [34]. Upregulated/downregulated genes were selected if their expression values exceeded the threshold of 0.05 FDR (Benjamini–Hochberg (BH) correction). Regarding the comparisons involving the two ALK+ALCL samples, we observed a lack of statistical power due to the small sample size. Therefore, a threshold of 1.32 was used for the absolute log-fold-change (logFC) to identify upregulated/downregulated genes. Then the Ensembl gene labels were converted into gene symbols using the biomaRt R package [35].

Gene set enrichment analysis was performed with the enrichR package [36] by considering the libraries KEGG_2021_Human, GO_Cellular_Component_2021, GO_Biological_Process_2021, and GO_Molecular_Function_2021, and using the Reactome library [37]. The BH correction for multiple comparisons was applied to evaluate the statistical significance of the enriched terms.

### 2.7. Immunohistochemistry

Immunohistochemistry for WT1 (clone 6F-H2, Dako, Denmark), pan-TRK (clone EPR17341, Abcam, UK) and IRF4 (Clone MUM1p, Dako, Denmark) was performed on FFPE tissue sections of 8 BI-ALCL, 4 cALCL and 2 ALK+ sALCL cases using an HRP-labeled Polymer Detection System (Envision System, Agilent Technologies) and an automated immunostainer (Omnis, Agilent Technologies for WT1 and IRF4; Autostainer, Dako for pan-TRK). Peripheral nerve tissue sections were used as a positive control for pan-TRK staining.

### 2.8. Cytogenetic Analysis

Primary tumor cells derived from a peri-implant breast seroma of patient #3, diagnosed with BI-ALCL at Sant’Andrea Hospital, Rome, Italy, were maintained in suspension culture in complete medium for 48 h (RPMI-1640 with 10% fetal bovine serum, 100 U/mL penicillin and 100 ug/mL streptomycin), supplemented with 50 IU/mL of recombinant IL-2 (R&D Systems, Minneapolis, MN, USA). Metaphases were harvested by adding colcemid for 30 min, followed by hypotonic KC1 treatment for 20 min and fixation (3:1 analar methanol: glacial acetic acid). After standard trypsin–Wright G-banding (GTW) and Quinacrine Q-banding (QFQ), the chromosomes were visually analyzed for abnormalities.

### 2.9. Targeted Next-Generation Sequencing

Genomic DNA was extracted from FFPE tissues of BI-ALCL tumor sample #3 and paired peripheral blood, using a PureLink^®^ Genomic DNA Mini Kit (Thermo Fisher Scientific, Waltham, MA, USA) and quantified using a Qubit ds DNA HS Assay Kit on Qubit 2.0 Fluorimeter (Invitrogen, Waltham, MA, USA) according to the manufacturer’s instructions. Next-generation sequencing (NGS) analysis was undertaken using a custom 26-cancer-susceptibility-gene panel related to DNA damage repair and cell cycle control (APC, ATM, BRD1, BRIP1, CDH1, CDK4, CDKN2A, CHEK2, EPCAM, MLH1, MRE11, MSH2, MSH6, MUTYH, NBN, PALB2, PMS2, PTEN, RAD50, RAD51C, RAD51D, RECQL1, SMAD4, STK11 and TP53) on the Ion Personal Genome Machine (Ion PGM™) platform (Thermo Fisher Scientific, Carlsbad, CA, USA). The panel contained 610 primer pairs in two pools, covering the exons and exon-intron boundaries. According to the manufacturer’s protocol, libraries were prepared by means of emulsion PCR using an Ion PGM™ Hi-Q™ View OT2 Kit on an Ion OneTouch 2 Instrument and the Ion OneTouch ES (Enrichment System) (Thermo Fisher Scientific) to produce high-quality Ion Sphere™ particles for use in combination with the Ion PGM™ Hi-Q™ View Sequencing Kit. The prepared libraries were sequenced on the Ion Personal Genome Machine (Ion PGM™) (Thermo Fisher Scientific, Carlsbad, CA, USA), using the Ion 318™ Chip v2 BC. Sequencing data analysis was performed using Torrent Suite version 5.0.5 and Ion Reporter version 5.16 (Thermo Fisher Scientific). PGM sequencing produced an average of 1,300,000 reads per sample, with the mean read length being 194 bp. The average read depth per sample was 2120 reads, with the mean percentage of reads on target of 95%. The mean percentage of regions of interest (ROI), with at least 500× coverage, was 97.5%, and with a uniformity of 97.67%. Somatic mutations were considered if the variant allele was present in more than 5% of the reads, considering a minimum coverage depth of 1000×. Called variants were imported, annotated and filtered in Ion Reporter Server (IRS). To reduce the effect of deamination in low-quality FFPE samples, the transition/transversion ratio (Ts/Tv) was calculated using bioinformatic tools in IRS and all identified variants were filtered. We used somatic mutation callsets to select candidate variant genes. This filter chain returned results for somatic SNVs based on the dbSNP, 5000Exomes, ExAC and UCSC Common SNPs annotation source databases. Allele frequency information for filtering true somatic variants with a minimum minor allele frequency ranged between 0.0 and >0.0001 in gnomAD. This filtering method also excluded variants of homopolymer lengths >7 with coverage lower than 500×.

## 3. Results

### 3.1. Fusion Transcripts in cALCL and BI-ALCL Are Uncommon and May Suggest Genomic Instability in BI-ALCL

**Figure 1:**
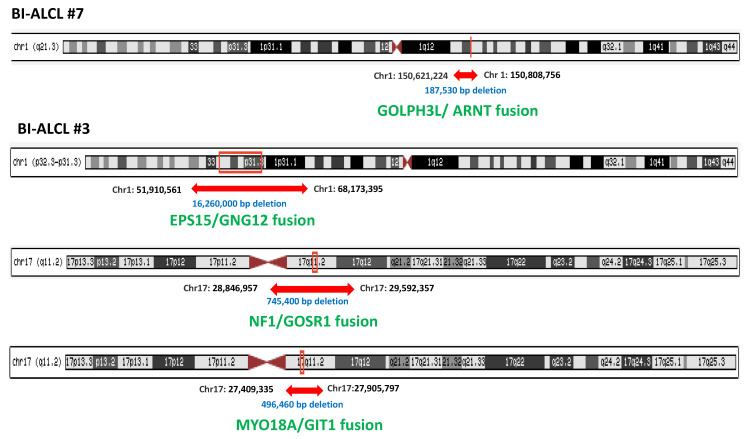
Intrachromosomal deletions in two cases of BI-ALCL resulting in the generation of fusion transcripts.

To evaluate the presence of fusion transcripts, we performed RNA-sequencing of 1385 cancer genes using FFPE samples of 12 cALCLs and 10 BI-ALCLs. Two anaplastic lymphoma kinase (ALK)-positive sALCL cases were included as controls, as this entity is characterized by driver translocations of the ALK gene. Only fusion transcripts detected using both software tools (FusionCatcher and FusionMap) and further confirmed via real-time PCR were analyzed in this study. NMP1-ALK rearrangements were detected in the two ALK+sALCLs (2/2, 100%), whereas the previously reported TYK2-NPM1 fusion was found in one cALCL (1/12, 8%) [5,38] (Table 2). None of the samples showed rearrangements of DUSP22, IRF4 or TP63 genes, even though high levels of DUSP22 and IRF4 mRNA, as well as intense nuclear staining for IRF4, were observed in the majority of both cALCL and BI-ALCL samples (Supplementary Figure S1 and Supplementary Table S1). This is in line with the observation that IRF4 protein expression in ALCL does not correlate with genetic alterations and is observed in cases with and without IRF4 gene rearrangement or extra copies [39]. Four different intrachromosomal chimeric transcripts were found in two out of 10 BI-ALCLs (20%). Two of them involved genes on chromosome 1 (EPS15-GNG12 and ARNT-GOLPH3L), whereas the other two mapped to chromosome 17 (MYO18A-GIT1 and NF1-GOSR1) (Table 2 and Figure 1).

**Figure 2:**
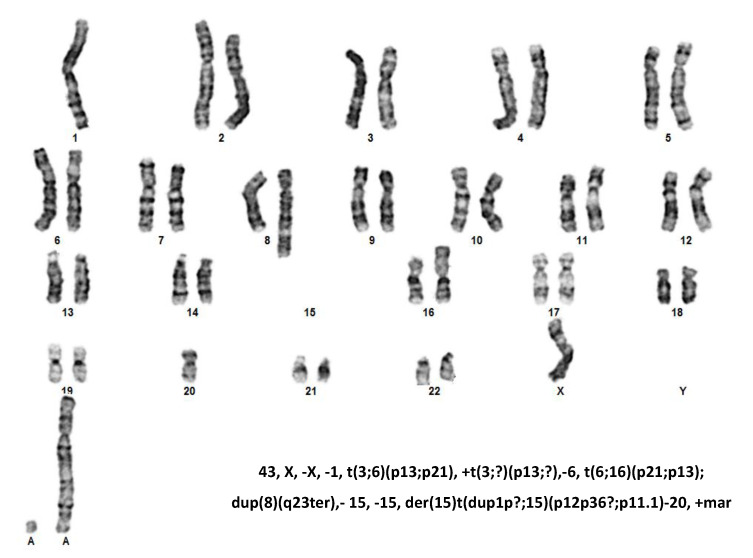
Karyotype of BI-ALCL #3.

Of note, three out of the four RNA fusions occurred in the same sample (BI-ALCL#3), which was also found to display a complex karyotype (Figure 2) including a deletion of chromosome 20, as previously reported in 66% of BI-ALCLs [22]. Cells deficient in certain DNA repair pathways can still survive by compensating the defect using error-prone repair pathways, which in turn favor additional mutations or chromosomal rearrangements, increasing genomic instability. To test the hypothesis that the high rate of genomic alterations found in our BI-ALCL sample could be related to mutations in genes involved in DNA damage repair and cell cycle control, we performed a targeted NGS with 26 cancer susceptibility genes, which revealed a germline variant of uncertain significance V1027L of the mismatch repair gene MSH6 (allele variant 49.80%). These data reveal that fusion transcripts are infrequent in both cALCL and BI-ALCL, and that in the latter they may occur as a consequence of intra-chromosomal deletions, possibly associated with genomic instability.

### 3.2. cALCL and BI-ALCL Show Upregulation of the PI3K/Akt Pathway and Differences in Transcription and Spicing Processes

**Figure 3:**
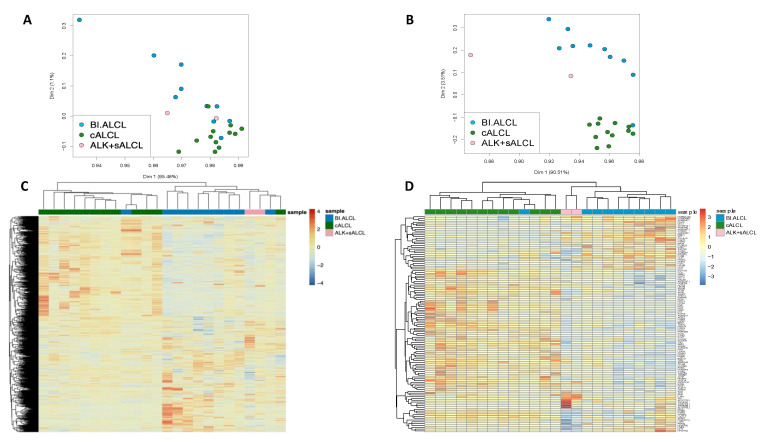
Unsupervised and supervised analyses of cALCLs, BI-ALCLs and ALK+ sALCLs. Principal component analysis (**A**,**B**) and hierarchical clustering (**C**,**D**) of all entities by cell type ((**B**,**D**) one-way ANOVA (BH-adjusted *p*-values < 0.05)).

Using the gene expression profiling data obtained via targeted RNA sequencing, we investigated transcriptional differences between cALCLs, BI-ALCLs and ALK+ sALCLs. We first performed unsupervised clustering, which showed cALCLs, BI-ALCLs and ALK+ sALCLs to be distinct according to both principal component analysis and hierarchical clustering (Figure 3A–C). Then we performed ANOVA analysis to highlight the genes that strongly discriminated the three groups (ANOVA, *p* value < 0.05, BH correction) (Figure 3B–D).

**Figure 4:**
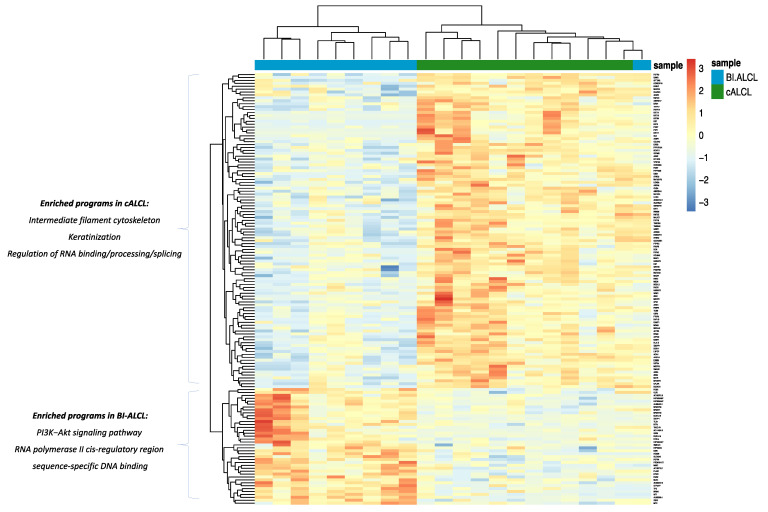
Heatmap showing differentially expressed genes and programs between BI-ALCLs and cALCLs.

**Figure 5:**
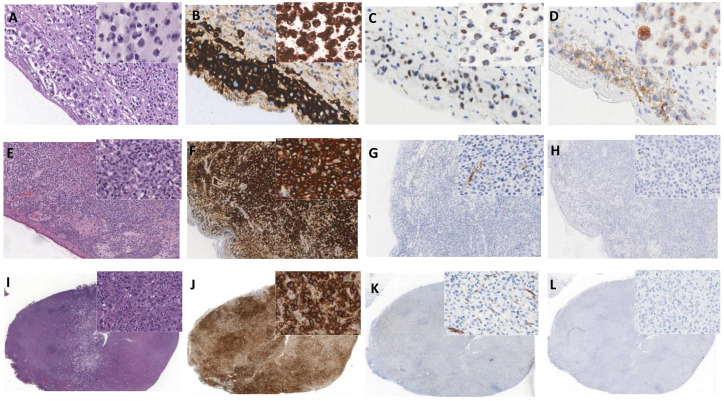
Immunohistochemistry of BI-ALCLs (**A**–**D**), cALCLs (**E**–**H**) and ALK+ sALCLs (**I**–**L**) for CD30 (**B**,**F**,**J**), WT1 (**C**,**G**,**K**) and pan-TRK (**D**,**H**,**L**). BI-ALCL tumor cells show nuclear staining for WT1 and membranous and cytoplasmic dot-like staining for pan-TRK in peri-implant breast capsular tissue (large panels, original magnification ×400) and peri-implant breast effusion (inserts, original magnification ×400). cALCLs (large panels, original magnification ×100, insets ×400) and ALK+ sALCLs (large panels, original magnification ×20, insets ×400) were negative for both WT1 and pan-TRK.

Among the 148 differentially expressed genes (DEGs) (*t*-test, BH-adjusted *p* value < 0.05) (40 genes upregulated in BI-ALCLs and 108 upregulated in cALCLs) that neatly separated BI-ALCLs and cALCLs (Figure 4, Table S2), WT1 was among the top-10 upregulated DEGs in BI-ALCLs (*p* = 9.1 × 10^−5^, fold change 4.78). To validate this at the protein level, we immunostained eight BI-ALCL, four cALCL and two ALK+ sALCL samples for WT1. In all eight BI-ALCLs, most of the tumor cells showed nuclear and occasional cytoplasmic staining, whereas the cALCL and ALK+ sALCL samples were negative (Figure 5).

Gene ontology (GO) and pathway enrichment analyses revealed that the most overrepresented GO terms and pathways in the BI-ALCL vs cALCL comparisons were related to the PI3K/Akt signaling pathway (upregulated DEGs: HSP90AA1, MYC, NTF3, FGFR4, EPOR) and to RNA polymerase II cis-regulatory region sequence-specific DNA binding (upregulated DEGs: MUC1, EGR2, NR6A1, HOXB9, TLX3, PLAG1, WT1, MYC, FOSB) (Figure 4, Table S3). In cALCL vs. BI-ALCL comparisons, overrepresented GO terms were related to intermediate filament cytoskeleton organization (upregulated DEGs: DSP, MACF1, DST, KRT17, KRT16, KRT14, PKP1, KRT5, SYNE2), likely due to the presence of contaminating cutaneous epithelial cells, and to RNA binding, processing and splicing processes (upregulated DEGs: DDX17, SRRM2, SCAF11, SNRNP70, HNRNPH1, HNRNPD, HNRNPR, PRPF8) (Table S3).

**Figure 6:**
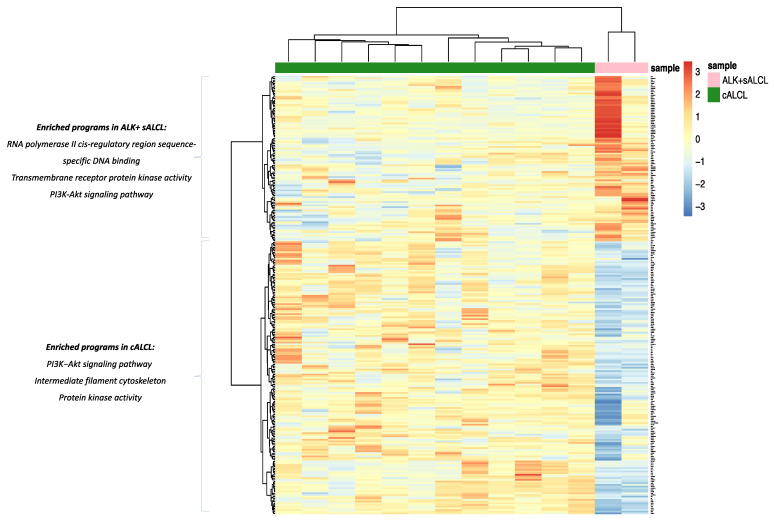
Heatmap showing differentially expressed genes and programs between cALCLs and ALK+ sALCLs.

An absolute fold-change threshold of 2.5 was considered to compare ALK+ sALCLs. In particular, we found 262 differentially expressed genes between ALK+ sALCL and cALCL (163 genes overexpressed in cALCLs and 99 genes overexpressed in ALK+ sALCLs) (Figure 6, Table S4). ALK was one of the most-overexpressed genes in ALK+ sALCLs. Among the gene programs enriched in ALK+ sALCLs were transmembrane receptor protein kinase activity (upregulated genes: ALK, RET, FGFR4) and RNA polymerase II cis-regulatory region sequence-specific DNA binding (upregulated genes: EGR1, DMRT1, BCL11A, CDX1, EBF4, PAX5, DACH2, GLI1, BACH2, FOSL1, MUC1, ZSCAN30, PAX7, MYB, PRDM16, FOSB, ZNF444, TEAD2, TEAD3). In addition to the intermediate filament cytoskeleton organization program, the PI3K/Akt pathway (upregulated genes: IL3, NTRK2, CCND2, YWHAQ, ERBB3, COL6A2, NTF3, FN1, NGF, FGFR3, FGFR2, EGFR) and the protein kinase activity pathway (driven by the highly expressed genes NTRK2, ERBB3, TYK2, FGFR3, FGFR2, EGFR, FRK, TTN) were also enriched in cALCLs (Table S5). Over-expression of TYK2 due to NPMN1/TYK2 fusion was identified in one of the cALCL cases (Table S6).

**Figure 7:**
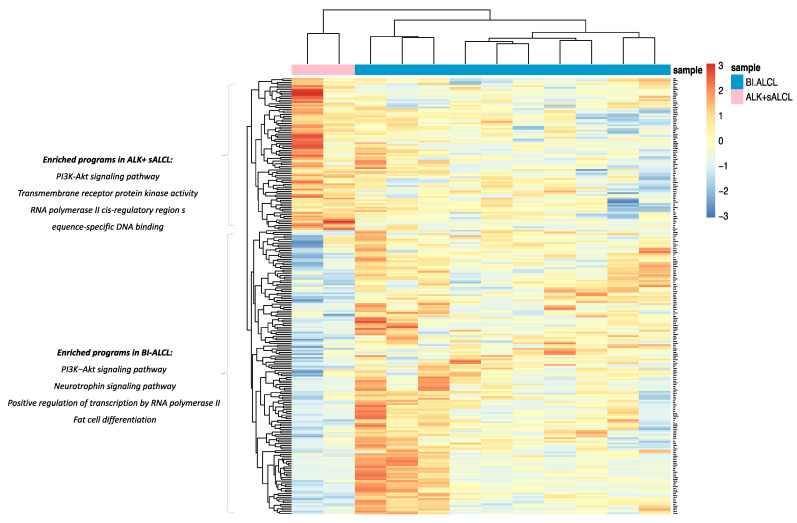
Heatmap showing differentially expressed genes and programs between BI-ALCLs and ALK+ sALCLs.

From the comparison of ALK+ sALCLs versus BI-ALCLs, we found 244 DEGs (159 genes upregulated in BI-ALCLs and 85 genes upregulated in ALK+ sALCLs) (Figure 7, Table S7). Again, ALK+ sALCLs showed enrichment in the RNA polymerase II cis-regulatory region sequence-specific DNA binding program and in transmembrane receptor protein kinase activity, with the latter program related to the over-expression of ALK, RET, PDGFRB and FLT3. The PI3K/Akt signaling pathway was enriched in genes such as PDGFRB, COL1A1, TCL1A, FLT3, IL2RA, MYB and IL2RB (Table S8). Conversely, in BI-ALCLs, the PI3K/Akt signaling pathway was enriched in different genes, such as NTRK1, IL3, NTRK2, FGF8, CCND2, ERBB3, NTF3, FOXO3, NGF, IL2 and FGFR2, Other over-represented GO terms in BI-ALCL were the neurotrophin signaling pathway (upregulated genes: NTRK1, NTRK2, NTF3, NGF), the positive regulation of transcription by RNA polymerase II, due to the over-expression of several homeobox genes (e.g., HOXC13, HOXD11, HOXA13, HOXA11, HOXA10), FOSB, ERG2, PAX3, LMO1 and WT1, and fat cell differentiation (upregulated DEGs: EGR2, HMGA2, PPARG, PPARGC1A).

Based on the described analyses, all the three types of ALCLs showed enrichment in the PI3K/Akt pathway though the upregulation of several genes, with similarities among BI-ALCLs and cALCLs, but due to divergent transcriptional modules/circuits in ALK+ sALCLs.

**Figure 8:**
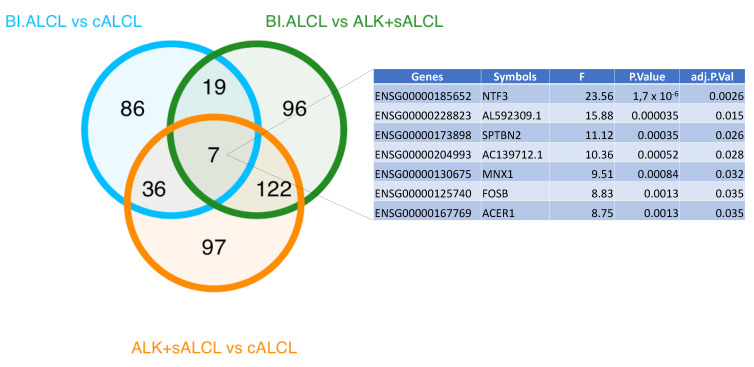
Venn diagram of the differentially expressed genes between BI-ALCLs, cALCLs and ALK+ sALCLs.

By comparing BI-ALCLs, cALCLs and ALK+ sALCLs, we found 120 DEGs, and among these, a differentially-expressed seven-gene signature that successfully discriminated among the three different types of ALCL (Figure 8). To validate the signature, we focused on the NTF3-Trk axis, since NTF3 and NTRK2 (encoding Trk-B) were overexpressed in BI-ALCLs and cALCLs. We therefore immunostained six BI-ALCLs, four cALCLs and two ALK+ sALCLs with a pan-TRK antibody which recognizes the C terminal domain of Trk-B, in addition to the Trk-A and Trk-C proteins (encoded by the NTRK1/3 genes). A membranous and occasional cytoplasmic dot-like staining was observed in the majority of tumor cells of BI-ALCLs, whereas all the other ALCLs were negative.

## 4. Discussion

The generation of fusion transcripts due to gene rearrangements represents one of the pathological mechanisms driving tumorigenesis in many hematological malignancies [40]. In peripheral T-cell lymphomas, ALK rearrangements with NPM1 and other genetic partners are the most common [41]. In ALK-ALCLs, whether systemic or primary cutaneous, next-generation sequencing of mate-pair genomic DNA libraries has identified two recurrent chromosomal rearrangements involving the DUSP22-IRF4 locus and the TP63 gene [10,11,12,13,14,15,16,17]. However, neither have been detected in BI-ALCL samples [18,42]. To further investigate the presence of fusion transcripts in BI-ALCLs and cALCLs, we pursued an RNA-seq approach using a pan-cancer panel and two different computational algorithms.

Our results show that fusions are relatively infrequent events in both BI-ALCLs and cALCLs. Indeed, we found a chimeric gene involving NPM1 (5q35) and TYK2 (19p13) in 1/12 cALCLs (8%). Similarly, Velusamy et al. identified TYK2 rearrangements in 4/32 (12.5%) cALCLs, including one with NPM1 as the 5′ partner gene. The NPM1-TYK2 fusion protein was found to activate STAT1/3/5 signaling [5]. Of note, we found TYK2 among the upregulated genes in cALCLs versus ALK+ sALCLs, which was associated with an enrichment in the protein tyrosine kinase activity program.

In BI-ALCLs we detected four different intrachromosomal rearrangements on chr. 1 (EPS15/GNG12 and ARNT/GOLPH3L) and on chr. 17 (MYO18A/GIT1 and NF1/GOSR1). All of the gene fusions were in-frame; however, their pathogenetic role could not be envisaged in the absence of functional studies. Indeed, many of the gene fusions detected via deep sequencing may represent epiphenomena of chromosomal instability [43]. In keeping with this possibility, three out of the four chimeric transcripts observed occurred in a single BI-ALCL that also showed a complex karyotype consistent with an unstable genome. Complex chromosomal abnormalities have been reported in other published BI-ALCL cases, but the underlying mechanisms of aneuploidy are not known [44,45,46]. Nevertheless, the NF1/GOSR1 fusion detected in one BI-ALCL is predicted to impair the open reading frame of the NF1 gene. Similar NF1/GOSR1 gene rearrangements causing inactivation of the NF1 gene have also been reported in lung cancer, where they are thought to play a role in the onset of adenocarcinomas lacking known driver mutations [47]. Indeed, neurofibromin (NF1) is a member of the mammalian Ras-GTPase-activating protein (GAP)-related proteins that promotes the conversion of active RAS-GTP into its inactive RAS-GDP state. Any loss of neurofibromin functionality will therefore result in prolonged activation of the RAS/RAF/MAPK and PI3K/Akt/mTOR signaling pathways and increased cellular proliferation and survival [48].

Recently, Lobello et al., showed a significant enrichment of mutated genes involved in the JAK/ STAT (*p* < 0.003) and PI3K/Akt signaling pathways (*p* < 0.02) in ALK− sALCLs compared to ALK+ sALCLs [49]. Our results indicate that BI-ALCLs, cALCLs and ALK+ sALCLs upregulate the PI3K/Akt pathway, but via different genetic pathways (Supplementary Tables S3, S5 and S8. In particular, ALK+ sALCLs showed enrichment in PDGFRB, FLT3 and IL2RA genes. PDGFRB and IL2RA have also previously been reported to be expressed in ALK+ sALCLs [50,51]. Conversely, in BI-ALCLs and cALCLs there was an enrichment in genes involved in the neurotrophin signaling pathway (NTRK1, NTRK2, NTF3, NGF), which in turn activates the JAK/STAT, PI3K/Akt and MEK/ERK signaling cascades to promote cell proliferation, differentiation and survival [52,53]. On immunostaining, BI-ALCLs but not cALCLs (or ALK+ sALCLs) showed consistent membranous positivity with a pan-Trk antibody, suggesting that the neurotrophin signaling axis may be particularly active in BI-ALCLs. This finding may be of potential therapeutic relevance, since the FDA has approved targeted TRK inhibitors larotrectinib and entrectinib for tumors harboring NTRK oncogenic chimeras, resulting in constitutive activation or overexpression of this kinase [54]. Importantly, our data exclude gene fusions as the mechanism driving NTRK expression in BI-ALCLs.

Another biological process that was found to be upregulated in BI-ALCLs and ALK+ sALCLs was RNA polymerase II (Pol II) cis-regulatory region sequence-specific DNA binding. Pol II transcribes all the protein-coding genes and its activity is highly modulated by different transcription factors [55]. In BI-ALCLs, several transcription factors were upregulated, including FOSB, WT1 and EGR2. In particular, FOSB (FBJ murine osteosarcoma viral oncogene homolog B) encodes for a leucine zipper protein that can dimerize with proteins of the JUN family, thereby forming the transcription factor complex AP1. AP1 regulates gene expression in response to various stimuli such as cytokines, growth factors and stress signals. In ALK+ sALCLs AP1 fosters proliferation and survival via PI3K/Akt signaling and immune evasion by promoting program death-ligand 1 (PD-L1) transcription [56]. We have previously shown that PD-L1 expression, observed in more than 50% of BI-ALCL cases, was associated with copy number alterations at chromosome 9p24.1 in only 33% of the cases, suggesting the involvement of additional mechanisms in its upregulation [23]. Interestingly, in the study by Oishi and colleagues, activation of the AP1 family of transcription factors emerged as one of the top gene sets positively associated with BI-ALCL as compared to non-BI-ALCL tumors [8].

WT1 (Wilms tumor 1) is a tumor suppressor gene that encodes a zinc finger transcription factor known to be involved in at least two distinct cellular processes: transcriptional control and RNA metabolism, depending on which isoform is generated by the alternative splicing of WT1 RNA [57]. WT1 has been reported to be expressed in acute leukemias [58] and in several lymphomas, including ALK+ sALCLs (3/6, 50%), ALK− sALCLs (14/31, 45%) and cALCLs (1/6, 17%) [59]. In our case series, WT1 expression was detected in all eight BI-ALCLs but not in the two ALK+ sALCLs or the four cALCL samples. This discrepancy may reflect the small number of cases assessed in our study. Studies of larger number of ALCL tumors are warranted to determine the true frequency of WT1 expression in different disease subsets.

The early growth response factor 2 (EGR2) transcription factor regulates the expression of genes involved in Schwann cell myelination and adipogenesis [60]. In keeping with these functions, we noted the enrichment of ERG2 and overrepresentation of the fat cell differentiation program in BI-ALCLs compared to ALK+ sALCLs. BI-ALCL tumor cells often show cytoplasmic vacuoles, but it is not clear if they represent lipid droplets [61]. ERG2 also plays an important role in controlling autoimmunity and inflammation by suppressing Th17 differentiation [62,63] and in inducing anergy in murine T cells by inhibiting IL2 production [64]. We and others have found that BI-ALCL tumor cells exhibit an activated CD4+ transcriptional profile with T regulatory features due to an IL10-, IL6-, IL13- and Eotaxin-rich cytokine microenvironment [6,7,65].

## 5. Conclusions

Our study employing RNA sequencing and transcriptome analysis of BI-ALCLs and cALCLs indicates similarities and differences between the two entities. Fusion transcripts, as a consequence of genetic rearrangements, appear to be infrequent in both, but when present, they likely play roles in disease pathogenesis. Despite differences in their transcriptional profiles, we demonstrate upregulation of the PI3K/Akt pathway in both lymphomas, which is associated with an enrichment in neurotrophin signaling pathway genes.

## Figures and Tables

**Table 1:** RT-PCR primers and probes recognizing fusion transcripts.

Target	Forward 5′-3′	Reverse 5′-3′	Probe 5′-3′	Amplicon Size (bp)
NPM1-TYK2	GGAAAAGACTCAAAACCATC	CCAGGATCACTCAGCTTG	CCTGGTTCATGGTAATGTGTGTGGCCGGAA	130
MYO18A-GIT1	CAAGGCAGCTTCTGATGATGG	AGTATTCCGGGTTAACAGGCAG	TGGTGACAGAGCGCAGTGCCGTGCCCTT	242
GORS1-NF1	TTTCTACCAAGCTGGGACTTCC	CCCAATAACACCCCCTAGGATG	GGATCAACCTGAGGAAGCGGCGGGACTCGC	229

**Table 2:** Fusion transcripts in cALCLs and BI-ALCLs.

Sample ID	Fusion Transcript	Genomic Location	Frame
ALK+ sALCL #1	NPM1/ALK	t(2;5)(p23;q35)	In-frame
ALK+ sALCL #2	NPM1/ALK	t(2;5)(p23;q35)	In-frame
cALCL #4	NPM1/TYK2	t(5;19)(q35;p13)	In-frame
BI-ALCL #3	MYO18A/GIT1	chr17(q11.2)	In-frame
	EPS15/GNG12	chr1(p32.3-p31.3)	In-frame
	NF1/GOSR1	chr17(q11.2)	In-frame
BI-ALCL #7	ARNT/GOLPH3L	chr1(q21.3)	In-frame

## Data Availability

Sequencing data are openly available in Sequence Read Archive SRA at https://www.ncbi.nlm.nih.gov/sra (accessed on 1 December 2021) (submission number SUB10353660).

## Supplementary Materials

The following are available online at https://www.mdpi.com/article/10.3390/cancers13246174/s1, Table S1: Clinico-pathological features of cALCL and BI-ALCL; Table S2: Differential expressed genes in BI-ALCL vs cALCL; Table S3: Enrichment analysis of UP- and DOWN regulated genes in BI-ALCL vs cALCL; Table S4: Differential expressed genes in cALCL vs ALK+sALCL; Table S5: Enrichment analysis of UP- and DOWN regulated genes in cALCL vs ALK+sALCL; Table S6. TYK2 expression levels in cALCL; Table S7: Differential expressed genes in BI-ALCL vs ALK+sALCL; Table S8: Enrichment analysis of UP- and DOWN regulated genes in BI-ALCL vs ALK+sALCL. Figure S1: DUSP22 and IRF4 expression in cALCL and BI-ALCL.
