# Supplementary material for: RNA Sequencing of Primary Cutaneous and Breast-Implant Associated Anaplastic Large Cell Lymphomas Reveals Infrequent Fusion Transcripts and Upregulation of PI3K/AKT Signaling via Neurotrophin Pathway Genes
Source: Cancers (Basel). 2021 Dec 7;13(24):6174. doi: 10.3390/cancers13246174 (PMC8699465; doi:10.3390/cancers13246174)
Supplement: Supplementary file 1 [file cancers-13-06174-s001.zip › cancers-1403324-supplementary- send to xml/cancers-1403324-Figure S1.pdf]

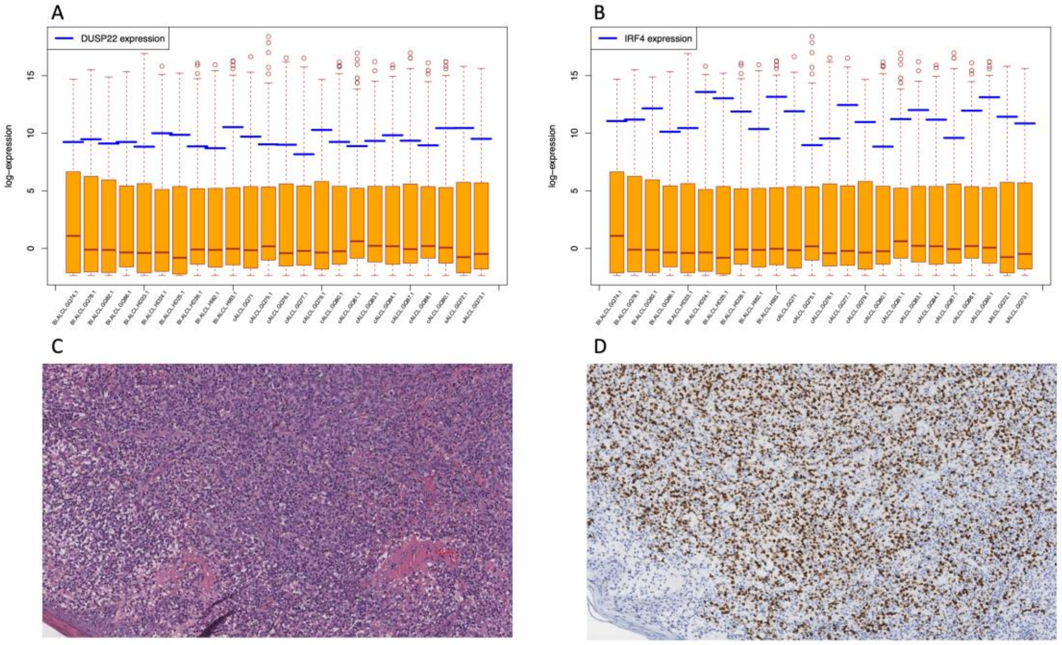

Figure S1. DUSP22 and IRF4 expression in cALCL and BI-ALCL. The expression levels of DUSP22 and IRF4 gene transcripts in the profiled samples (A, B) are highlighted by the blue segments in the log-expression box plots in which the median expression of the entire set of genes analyzed by RNAseq is indicated by the red bar. The plots show that both DUSP22 and IRF4 transcripts are consistently and rather homogeneously expressed in the samples with no relevant variations between the different entities. Representative image of IRF4 protein nuclear expression in a cALCL without IRF4 rearrangements (C: haematoxylin and eosin, original magnification x100; D: IRF4, original magnification x100).
